# Centering communities in policy implementation research: considerations for the integration of community-based participatory research principles into policy-focused implementation science

**DOI:** 10.1093/tbm/ibag057

**Published:** 2026-09-09

**Authors:** Rajinder Sonia Singh, Sara J Landes, Maji Hailemariam, Matthew Lee, Ana M Progovac

**Affiliations:** Center for Mental Healthcare and Outcomes Research, Central Arkansas Veterans Healthcare System, Little Rock, AR, United States; Department of Psychiatry, University of Arkansas for Medical Sciences, Little Rock, AR, United States; Behavioral Health Quality Enhancement Research Initiative (QUERI), Central Arkansas Veterans Healthcare System, Little Rock, AR, United States; Department of Psychiatry, University of Arkansas for Medical Sciences, Little Rock, AR, United States; Behavioral Health Quality Enhancement Research Initiative (QUERI), Central Arkansas Veterans Healthcare System, Little Rock, AR, United States; Charles Stewart Mott Department of Public Health, College of Human Medicine, Michigan State University, Flint, MI, United States; Department of Obstetrics, Gynecology and Reproductive Biology, College of Human Medicine, East Lansing, MI, United States; Department of Population Health, NYU Grossman School of Medicine, New York, NY, United States; Department of Population Health, NYU Grossman School of Medicine, New York, NY, United States; Institute for Excellence in Health Equity, NYU Grossman School of Medicine, New York, NY, United States

**Keywords:** policy implementation, community-based participatory research, implementation science, community-engaged dissemination and implementation science

## Abstract

**Background:**

Community-engaged dissemination and implementation research focuses on the implementation of evidence-based interventions within clinical or community-based settings. It uses community-engaged processes or partnerships. This includes (but is not limited to) community-based participatory research (CBPR). CBPR is an approach that centers actively engaging and collaboratively sharing power with community members, which can increase the value of studies for the communities being served and researchers. Community members include those who are most affected by the research (e.g. patients, citizens) and those most invested in the communities being researched (e.g. advocates, leaders).

**Main body:**

Policy implementation research is the subfield of implementation science that aims to understand how policies are implemented and improve their implementation. Previous literature discusses policy co-design, defined as actively partnering with community members in the policy making process. In this article, we suggest ways to integrate CBPR methods within policy *implementation* research as well. We specifically discuss ways that community-members can be actively and substantively engaged in policy implementation in addition to policy making.

**Conclusion:**

Policy implementation research researchers who wish to approach their work through a CBPR lens would benefit from our overarching recommendation to meaningfully engage community members throughout the policy implementation research process including (i) building or maintaining meaningful partnerships, (ii) engaging in transparent and equitable resource sharing, cost charging, compensation, and incentives. (iii) co-designing/co-conceptualizing projects, (iv) co-refining the project and co-selecting strategies for project completion, (v) bi-directional learning and open channels of communication during the project period, and (vi) taking action on harmful or inequitable policy implementation research and results of research. We argue that if policies are causing harm or not reaching marginalized communities, researchers should alert policymakers, frontline staff, and community members to this information and actively assist in providing solutions to this issue. Ideally, the steps outlined in this article will enable researchers to directly uncover the presence of harm and/or lack of reach of policies and form the communication channels that would enable researchers to inform policymakers and community members.

## Teaser

Policies are often developed without underserved communities in mind. The current paper provides recommendations to actively partner with underserved communities when developing, carrying out, and measuring the impact of a policy that is being implemented.

Implications **Practice:** Integrating community-based participatory principles into policy implementation practices may assist implementation practitioners with increasing community engagement and ensuring policies are delivered equitably.

**Policy:** The current work provides recommendations to assist with integrating community-based participatory principles into policy-focused implementation research which may increase the likelihood that policies are researched and delivered equitably.

**Research:** Policy-focused implementation research may benefit from considering how to integrate community-based participatory principles to ensure communities of interest are actively engaged throughout the research process, including from planning the research project to dissemination of results.

## Background

The field of implementation science is currently moving toward an active focus on health equity [1, 2]. Meaningfully and substantively partnering with community members in implementation science can improve access to and quality of innovation delivery for marginalized communities [3]. Actively partnering with community members in research can also increase retention of research participants, improve fidelity, and improve outcomes among marginalized communities [3]. These improvements in research and health outcomes through the active partnering with community members are known as community-based participatory research (CBPR) [3, 4]. CBPR includes partnerships between important key collaborators such as community members, researchers, policy makers, and others [3, 4]. There are currently frameworks and strategies for policy co-design [5], and frameworks for policy dissemination and implementation (e.g. Bullock’s integrated determinant’s and process implementation framework [6], the Framework for Dissemination of Evidence-Based Policy) [7]. However, guidance for co-designing implementation of existing policies is limited. This article provides recommendations for centering those most impacted by specific policies by integrating CBPR principles into policy implementation research.

## Implementation science and health equity

Implementation science may improve use of a practice with some segments within a setting, but may not impact all individuals evenly, thus potentially contributing to or worsening a disparity [8]. Implementation science can improve health equity when the implementation effort for the evidence-based practice concretely focuses on addressing health disparities, and the evidence-based practice is implemented in a setting with explicit consideration of marginalized communities [2].

One research approach that improves health equity is CBPR which involves shared decision-making [9, 10]. CBPR is most common in health disparities research to highlight the needs of specific communities and decrease disparities with solutions that are acceptable to these communities [9, 10]. Building and sustaining meaningful partnerships with key collaborators on the ground (e.g. community members/organizations) enhances the likelihood of success [9]. Engaging community members can help identify opportunities for study results to be used effectively to create positive impacts on health for marginalized communities [9, 10].

There are calls to integrate CBPR and implementation science, mainly acknowledging the shared goal of (i) reducing the research-to-practice gap and (ii) co-creating knowledge [11, 12]. In response to calls to integrate implementation science and CBPR, researchers have developed or refined different approaches to engage community members in implementation work. Some examples include user-centered design, direct to consumer marketing, and the newly designed Engaging All Voices (formerly Consumer Voice) toolkit which guidance and tools to engage community members [11, 13]. The integration of CBPR into implementation science in general is growing. Our hope is to describe how these methods can be applied to *policy* implementation research specifically.

## Policy implementation research and practice

Implementation scientists can conceptualize policy in four ways including (i) policy as something to adopt, (ii) policy as something to implement, (iii) policy as context to understand, and (iv) policy as a strategy to use [14]. In the current paper, we are focusing on (ii) policy as something to implement and research when implementing policy. Policy implementation researchers aim to improve the implementation of existing policies and optimize existing policy delivery to improve health benefits [15]. Policy implementation research is focused on understanding how entities who pass policies put them into effect [15]. In policy implementation research, there are two broad categories of study including policy *process* implementation studies and policy *impact* implementation studies [15]. Policy process implementation studies document how the policy was implemented [15]. Policy impact implementation studies use experimental or quasi-experimental designs to assess outcomes related to effectiveness or implementation of the policy [15].

While the integration of CBPR into policy implementation is a new approach, there is literature describing policy co-design—a strategy for actively engaging community members in the policy making process [5]. Policy co-design strategies offer a roadmap to engage community members in the process of creating policies [5]. We expand on this work and discuss how to integrate CBPR and policy implementation methods.

## Recommendations for integrating CBPR principles into policy implementation research

Given the focus of CBPR on moving traditional, non-participatory research into policies to improve health for marginalized communities, a combination of taking a CBPR approach and policy implementation research may be beneficial. This integration of approaches would allow for research conducted in partnership with communities to be translated into policies *and* improve the equitable implementation of those policies. We reviewed several existing CBPR frameworks and tools for implementation science [3, 11, 13, 16–19]. Based identified shared constructs and unique contributions, we offer suggestions (Table 1) for policy implementation researchers, specifically academics or investigators conducting policy implementation projects and evaluations [2, 3, 11, 17–22]. For each recommendation, we created real-world hypothetical vignettes to provide tangible examples of how to apply our recommendations in practice and how applying these principles can alter a policy implementation process.

**Table 1:** Recommendations for integrating CBPR methods into policy implementation projects.

Recommendation	Examples of how to operationalize recommendation
Building or maintaining meaningful partnerships	Obtain formal and informal training and mentorship in best practice for community-engaged research prior to engaging community members Invite community members through organizations serving that community or local advertisements Prepare and share accessible materials describing research team members and project Allow for several months to meet with community members, answering any questions they may have, Understand community members’ needs (i.e. outputs) from the research project and create a timeline for delivering outputs Inform community members about timeline of research project and potential delays. Consider inviting a community member to serve as co-lead on project Develop an agreed upon plan for communication and a method to ensure everyone’s feedback is considered Engage in ongoing co-learning exercises between community members and the research team Solicit feedback from community members of the research team throughout the policy process evaluation process Inquire with community members if there are pieces of context the research team may be missing or not considering that is important to the community Invite community members to participate in dissemination efforts Involve community members in the design and evaluation of the policy impact evaluation Include community members in the evaluation in ways that are acceptable and appropriate to them Affiliate the community member with the institution so they may have access to de-identified data and access to shared network drives
Engaging in transparent and equitable resource sharing, cost charging, compensation, and incentives	Ask community members how they would prefer to be compensated If funds are not available, consider volunteering time in a way that is helpful to the community (e.g. giving a presentation, participating in a community event) Inform community members about process of and timeline to payment and approximately how long it may take for payment to be received Compensation may also take the form of assisting the community member with their curriculum vita, biographical sketch, other support document, and/or resume to build their capacity for conducting research or other academic endeavors Co-create a memorandum of understanding with clear expectations for researchers and community Provide compensation for research team meetings attended and research activities performed that are aligned with the amount of time spent on the project
Co-designing/co-conceptualizing the project	Ask community members if a key perspective is missing from the project Ask community members about their awareness of the policy and how it is being implemented, If community members are not aware of policy, provide information about policy and plans for implementation project Inquire about potential concerns or thoughts Assess the needs of the community and which impacts are most important to them
Co-refining the project and co-selecting strategies for project completion	Ask community members about their preferred methods of communication throughout the project Ask community members about how to best track whether tasks are completed Collaboratively brainstorm how to solve problems that arise during the project Provide community members with information about implementation strategies Include community members in selection of implementation strategies Include community members in testing of implementation strategies
Bi-directional learning and open channels of communication during the project period	Co-create tools (e.g. fact sheets) and share with community organizations Provide presentations or infographics with research updates Provide feedback throughout the policy process evaluation at a cadence that is acceptable to the community organizations Conduct ongoing check-ins with community members Provide research project results to community organizations in a way that is accessible (e.g. zine, appropriate readability level)
Taking action on harmful or inequitable policy implementation research and results	If community members express concerns during planning process, consider methods for addressing their concerns in preparation for the project Inform research team and community organizations of harm caused Consider ways to change policy implementation research to alleviate harm or increase equitable implementation of policy Engage in shared advocacy with community members to change policy implementation efforts

### Preparing for policy implementation projects

Vignette 1 offers an example of using a CBPR approach when preparing for a policy implementation project. When preparing to conduct policy implementation projects or examining policy implementation determinants, we recommend inviting people from communities of interest to serve as members of the research team [13, 19]. This may include partnering with community organizations that serve community members. Some challenges to partnering include navigating limited connection to communities, timelines in research, and an understandable community distrust of researchers among marginalized communities [3]. To address the challenges of limited community connection and distrust, the research team will likely need to take time to engage the community as partners. When partnering, it is important to inquire about the community members’ needs, priorities, and assets and offer compensation to community members in ways that are acceptable to them. Compensation can include offering financial compensation, mentorship, presentations, or food. Asking how community members would like to be compensated and working within our power structures to do so establishes trust and demonstrates researchers’ interest in providing communities with what they need versus what we assume they will want [13]. If researchers do not have funds available to pay community members directly, compensation may include volunteering time in the form of providing a workshop or mentorship or connecting to institutional resources or knowledge systems (e.g. networking community partners with these systems). Building trust may include using departmental funds or donations to purchase items for the community. Another example of providing support and increasing capacity may be assisting community members with their biographical sketches to create capacity for additional research. It is important to spend time getting to know community members by attending community events or listening sessions prior to approaching with one’s research idea or need. Further, universities may consider adding structural value to researchers who actively partner with or engage communities.

To address the challenge of timelines, practice transparency by keeping an open dialogue and communication channel related timing of research proposals and the necessary approvals that may delay research projects [19]. If a situation arises where a community requires a product prior to research funding approval, it may be beneficial for researchers to consider applying for intramural funds with shorter timelines [17, 19]. These awards may be applied for with the researcher and a community member researcher co-serving as primary investigators.

When community members agree to be a part of the research team, researchers should consider how to engage in collaborative power sharing. With permission, all team members may consider sharing their individual positionality. It is also helpful to clearly outline roles and responsibilities, and group processes including decision making, co-creating products, and ownership of products. Groups should also consider how disagreements will be brought up and resolved (e.g. anonymous concern box) [22]. It is important to ensure all members have decision-making power on the project team [17, 18]. In practice, this may look like two project leads—the research principal investigator and the community member principal investigator [23]. If it is not possible to formally name them, then select a member or members of the community who may hold additional decision-making power or influence on the research process. Sometimes it may not be feasible or researchers may not have the power to act on suggestions from the community [19]. In this case, researchers may consider telling community members the limitations of their roles and collectively brainstorm how they may address the concerns of the community members (e.g. connect to other resources or organizations who may be able to assist or act on the suggestions).

Engaging communities may include co-creating tools (e.g. fact sheets) for policy implementation projects and actively seeking feedback on methods and plans for the project [23, 24]. Additionally, ask community members what they most want about the implementation of a particular policy and include their suggestions into the policy implementation project. Another suggestion is to expand the partnership with community members, which could include actively asking community members, “Who is not here who should be here? Whose voice may we be missing and how can we best include them?” [13].

Another suggestion is to work with community members to select policy implementation strategies that are acceptable to the community and feasible in the setting in which the project is being conducted [25]. When gathering information about barriers and facilitators to implementation of a particular policy (even if it is not focused on a particular marginalized population), it may also be helpful to assess if participants are aware of the policy and the potential impact it may have on marginalized populations. For example, a state-wide policy may be passed that provides vouchers for children to attend charter and private schools via a lottery system. However, this policy does so by redistributing funds from public schools. It includes a clause in which educators cannot teach about historical oppression of marginalized populations within the United States. Although the policy does not specifically focus on marginalized communities, it has a significant impact on those who are marginalized, so it would be helpful to ensure there are methods in place to measure the impact on marginalized populations.

### Conducting policy process evaluations

Vignette 2 offers an example of using a CBPR approach when preparing for a policy process evaluation. Policy process implementation studies describe or document how the policy was implemented [15]. We recommend assessing the impact of policies on marginalized populations before, during, and after they are created—especially if these policies are specific to a particular community of marginalized people [26]. In practice, assessing the impact of policies on marginalized communities could include asking if there are any members of the community the policy does not reach or may unintentionally harm and measuring if receipt is similar across demographic groups [27]. Even if the policy does not focus on a marginalized community, it is important to assess if the policy is equitable (i.e. are marginalized communities left out or further marginalized by this policy).

We encourage actively including and soliciting feedback from the community members who are a part of the research team and asking for feedback during the process evaluation that the researcher may not have considered [17, 19]. For example, community members may avoid settings where a policy is being implemented and the research team is unaware of this. An example of this may be a needle-exchange program offered in a particular area where community members are aware of an increased law enforcement presence. While the needle-exchange program is meant for harm reduction, community members may avoid the area out of fear of being arrested for possession of drugs.

Process evaluations are often conducted by the research team without feedback to others or changing the process. We argue that if findings indicate that policy implementation is actively harming or unhelpful to marginalized communities, it would be helpful to change the process of how the policy is implemented in the service of increasing equity [2]. This could include providing feedback to the policy makers through briefs and proposals, informing local community organizations about the results and organizing with them to make these changes, and informing organizations who may be tasked with implementing the policy. Providing this feedback at multiple levels allows for potential changes throughout the policy implementation process.

Challenges to conducting policy process implementation studies include logistical difficulties related to data ownership and data sharing as well as difficulties related to the needs of the funder. Often data are being collected from or by community organizations but belong to a healthcare system or institution that represents the researcher. One way to address this issue is to discuss with community members up front who owns the data and what processes are in place to protect the data [17, 19]. For example, if data regarding sensitive information are collected, inform them about steps to protect data. Researchers may consider the process to formally affiliate community members with the institution and/or listing community members as paid consultants on grant applications so they may have full access to data. This process may be cumbersome and timely, so beginning it during the start of the research process is ideal. The process of including community members as affiliates or consultants further substantively engages them in the research process. Actively including community members as a part of the research team will allow for sustained relationships with the communities and the research team.

### Conducting policy impact evaluations

Vignette 3 offers an example of using a CBPR approach when preparing for a policy impact evaluation. In preparing for the policy impact evaluation, we encourage gathering feedback from community members as discussed above. We encourage continued partnerships with community members to assess the needs of the community and which impacts are most important to them [17, 19]. This may include continuing partnerships after the study/funding has ended to provide feedback and potentially prepare action items with the community. We encourage reporting back findings to the community [17, 19]. Reporting findings to the community at large may include attending events hosted by community members or additional ways to disseminate findings (e.g. zines–a small published online or print work, websites, flyers) that can be easily accessible in communities [17, 19].

Conducting evaluations at the end of the study period presents a challenge because further marginalization or harm could be caused during the study period without detection [17, 19]. Similar to process evaluations, if the results indicate harm was caused to marginalized groups or policy implementation was not equitable for those groups, provide actionable items on changing policy implementation strategies or recommend changes to the organization responsible for the policy [2, 3, 11, 17–19]. If this project was conducted with a specific team of community members, at minimum researchers should inform the team of the harm caused and discuss different implementation strategies that are focused on equity. This process may include selecting specific implementation strategies that focus on a particular community of interest based on findings. If harm was caused as a result of the policy implementation efforts, engage in shared advocacy with community members to implementation efforts. If the policy itself was harmful, consider ways to organize with community members to advocate for policy change (e.g. testify for change).

In addition to providing feedback to community members, we recommend eliciting feedback on how policy implementation could be improved. We recommend including this feedback in relevant reports, dissemination efforts, and next steps to the organization or institution responsible for the policy and funders.

## Conclusion

**Figure 1:**
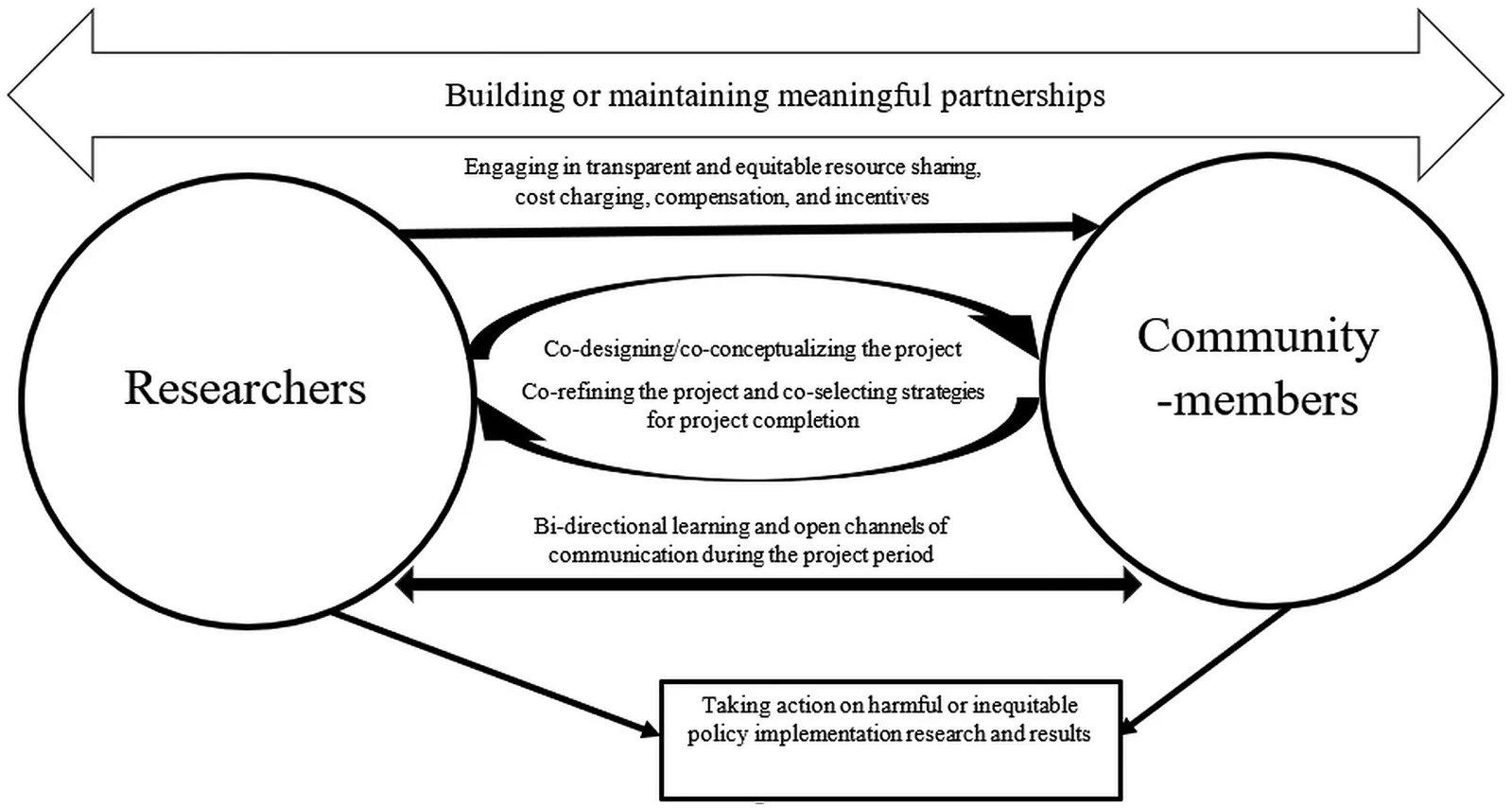
Visual of recommendations for integrating CBPR methods into policy implementation projects.

Implementation research programs utilizing CBPR approaches would benefit from our overarching recommendation to involve community members throughout the policy implementation research process including (i) building or maintaining meaningful partnerships; (ii) engaging in transparent and equitable resource sharing, cost charging, compensation, and incentives; (iii) co-designing/co-conceptualizing projects; (iv) co-refining the project and co-selecting strategies for project completion; (v) bi-directional learning and open channels of communication during the project period; and (vi) taking action on harmful or inequitable policy implementation research and results of research (Fig. 1). We argue that if policies are causing harm or not reaching marginalized communities, researchers should alert policymakers, frontline staff, and community members to this information and actively assist in providing solutions to this issue. Ideally, the steps outlined in this article will enable researchers to directly uncover the presence of harm and/or lack of reach of policies and form the communication channels that would enable researchers to inform policymakers and communities of interest. The recommendations outlined here are not exhaustive and include several bi-directional pathways of communication to build lasting, mutually beneficial partnerships to improve health for underserved communities.

## Data Availability

This is a commentary paper. No unique data were gathered for the paper.

## Welfare of animals

This article does not contain any studies with animals performed by any of the authors.

## Appendix 1

Case Vignette 1. CBPR in preparing for the policy implementation projects
**Dr J investigates low uptake of hormone treatment** 
**(1) Building or maintaining meaningful partnerships** Dr J is an early career researcher who is working in a low-cost, sliding-scale healthcare system with several clinics tasked with evaluating the implementation of a new state-wide policy to implement evidence-based care for transgender patients (hormone therapy for gender dysphoria) at a reduced cost if they meet certain criteria. There are health care experts in the system that are familiar with the evidence-based practice, but she is wondering if there are potentially pieces of the puzzle that may be missing because none of the project team members responsible for the implementation and evaluation of this policy are transgender or gender diverse.

- Obtaining formal and informal training and mentorship in best practice for community-engaged research prior to engaging community members

Dr J first begins taking courses on her own in learning CBPR online and reaches out to Dr W at a nearby university for mentorship. Dr J asks Dr W if she would be willing to meet with her biweekly for mentorship on the project in exchange for authorship on projects. Dr W agrees and begins providing Dr J with guidance on how to engage community members. Dr W encourages Dr J to reach out to local organizations to learn more about the organizations and find out if they would be interested in potentially partnering on the project.

- Inviting community members to join project efforts through organizations serving that community or local advertisements

Dr J looks through social media and search engines for local community groups for the transgender community. She sends those groups a blurb about herself, the state-wide policy (noting they may already be familiar with it), the healthcare system, and their project to implement and evaluate the implementation of the state-wide policy across their healthcare system. She states she is looking for a group of people to be a part of the project to ensure that the experiences of the people most affected by the policy (transgender people) are actively involved in the policy implementation efforts. She includes her email address and phone number and states that interested people can email, call, or text her. Prior to sending this blurb, she sends it to her mentor who sends it to a community member who is also a faculty member within her institution. The community member makes some changes and suggests that Dr J include her pronouns in the blurb. Dr J makes the suggested changes and emails the blurb to the organizations and asks if they would be willing to share the blurb with their members.

- Allow time to meet with community members, build rapport, answer questions, and clearly communicate about a potential project

Some organizations ask Dr J to come and speak in person. She attends their meetings and talks about herself and the project. Several interested community members in the different organizations contact Dr J. She sets up individual meetings with them to talk to them both personally and professionally. She discusses the timeline of the project, the goals of the project, and answers any questions community members may have. She also asks community members what they may wish to gain from being a part of the project team. She states they will also have these conversations later to ensure she continues to honor their preferences/their answers are the same or if anything has changed. Dr J has five community members with varying backgrounds (e.g. gender, race, socioeconomic status) who have agreed to be a part of the project. This ensures that she is not tokenizing one person to speak for the whole community. Dr J gives the community member team members the title of “Community Experts” for them to able to put the role on a resume or curriculum vitae and to provide them with power in the room during team meetings.
**(2) Engaging in transparent and equitable resource sharing, cost charging, compensation, and incentives** Dr J tells the Community Experts she has funds available to pay them $50 per hour to be a part of the project. This would require completing paperwork to receive direct deposits within the healthcare system. Dr J lets the Community Experts know that people often find this process frustrating and that some have decided not to accept payment because of it. She states that she and her research assistant are willing to help with the process, but she wants them to know up front about the frustration of completing the required paperwork.

- Ask community members how they would prefer to be compensated

Dr J asks if the monetary payment is reasonable or if there is another way the Community Experts would like to be compensated. One responds that he wishes to pursue graduate school, so he would prefer mentorship from Dr J and opportunities for research products. Three state that the monetary payment is acceptable. Another person states that she was hoping this would serve as her volunteer hours for her school program next fall and asks if Dr J could sign off on those hours. She also asks if Dr J would be willing to present the findings to the transgender health student organization that she runs in her school. Dr J indicates that she is willing to support in this way and also asks if any members of the team are also interested in presenting to the student organizations.
**(3) Co-designing/co-conceptualizing the project** The project team begins meeting to plan the project. In the first project team meeting, Dr J provides an overview of the state-wide policy. The administrative personnel are familiar with the policy and the criteria needed for patients to qualify. The health care personnel are familiar with the evidence-based practice.

- If community members are not aware of policy, provide information about policy, plans for implementation project, and inquire about potential concerns or thoughts

One Community Expert speaks up and states he had not heard of this policy change until Dr J presented at his community organization meeting. He imagines a lot of people have not heard about it either. Dr J asks if he has any concerns or thoughts about the state-wide policy. The Community Expert responds he thinks it is a great policy and he thinks a lot of people will qualify. He just wants to make sure that the word gets back out to people. Dr J asks if he has thoughts on how to get the word out. He responds likely social media, the organizations that Dr J emailed and presented at, and he also suggests local establishments that people in the transgender community visit in the city. Dr J follows his advice and uses these same avenues to create and disseminate an info sheet about the public policy.

- Actively ask community members if a key perspective is missing from the project

Dr J asks if there is anything missing from the project to the entire project team. After a number of members of the project team who are affiliated with the health care institution speak, Dr J specifically asks, “Community Experts, anything you all noticed that was missing?” In meetings where there may be perceived or actual power differentials, it can be beneficial to ensure that people have equal opportunities to provide their opinion. One community member speaks and states that she noticed there is not a section for a preferred name and pronouns in the electronic health record and that it is likely transgender patients would not be comfortable attending clinics for health care if they do not have a place to enter this information easily. Dr J thanks her for the contribution and it is integrated into the implementation planning guide to alter the electronic health record prior to implementation.
**(4) Co-refining the project and co-selecting strategies for project completion** Dr J wants to ensure that the implementation strategies that are selected for the project are acceptable to all members of the project team which includes researchers, healthcare system administration, health care providers, staff, and community members.

- Provide community members with information about implementation strategies

During one of the project team meetings, Dr J gives an overview of different policy implementation strategies. She also provides an overview of the Expert Recommendations for Implementing Change (ERIC) taxonomy. She reminds the project team that she will do this again later, but she wanted to introduce this now in case people had questions about them. She provides examples of studies that have used different policy implementation strategies to provide concrete examples of the strategies in action. She asks if anyone on the team has been involved in implementation efforts and to provide examples of the strategies they used to do so.
**(5) Bi-directional learning and open channels of communication during the project period** In addition to sharing the barriers and facilitators with the project team, Dr J wants to share the barriers and facilitators with the community organizations that she originally reached out to about the project to maintain partnerships and bi-directional learning. She brings the barriers and facilitators slide deck to a meeting and discusses with the team the best way to share it with community organizations.

- Co-creating tools and sharing with community organizations

One Community Expert suggests circulating a zine. Dr J asks, “What’s a zine?” The Community Experts explain that zines are self-published, brief booklets that offer information about a particular topic. Zines are especially popular in subculture or marginalized communities. Dr J and the Community Experts work together to co-design a zine about the state-wide policy initiative, barriers and facilitators to implementation in the healthcare system, and their next steps. They print 500 copies and distribute them at the local community organizations.
**(6) Taking action on harmful or inequitable policy implementation research and results** Toward the end of a project team meeting, Dr J asks if anyone else has any questions or concerns about the project. One of the Community Experts says that they have a concern that is not about the project, but is about the policy overall. They stated several weeks ago they had brought up that the electronic health record did not have a preferred name and pronoun field in the healthcare system. They noted it made them worry now how many other healthcare systems may not have these fields or other knowledge about transgender people and are being mandated to implement this state-wide policy.

- Engage in shared advocacy with community members to change policy implementation efforts

The project team in partnership with community organizations collaboratively drafted a letter to the legislator about recommending an addendum to the policy to ensure that the electronic health records include preferred name and pronouns or at a minimum that all of the healthcare systems in the state be recommended to have their employees complete training on delivering evidence-based practices for transgender patients prior to implementation of the state-wide policy. The letter included recommendations for who could provide this training.

Case Vignette 2. CBPR in conducting policy process evaluations
**Cross-collaboration to reduce health care disparities in measurement-based care** Dr R works in a national healthcare system that is implementing measurement-based care in their mental health clinics. Specifically, the healthcare system is requiring that every patient complete the General Anxiety Disorder-7 (GAD-7) and Patient Health Questionnaire-9 (PHQ-9) every two weeks and use these measures to guide shared decision making in mental health treatment. Dr R is tasked with leading the project team that is managing the implementation efforts. Four months into the implementation effort, the implementation team does a preliminary data analysis to examine any potential patterns. They realize that Black patients and Hispanic patients have large amounts of missing data. Dr R was unsure where to start as he was not familiar with health equity research and did not understand why this health disparity may be occurring. Another researcher in the system refers him to Dr A. Dr A has expertise in implementation science, CBPR, and has worked on several projects over the last 10 years on improving health equity for Black and Hispanic patients in the healthcare system.
**(1) Building or maintaining meaningful partnerships** Dr A is already well-partnered with several organizations that serve Black and Hispanic people who access care within the healthcare system. She works to leverage her existing partnerships with community members to understand the disparities and select policy implementation strategies to reduce the disparities. She connects four community members to the implementation team.

- Engage in ongoing co-learning exercises between community members and the project team

In order to recognize that everyone has something they have expertise in, each implementation team meeting starts with 5 minutes of “show and tell” where each person gets to share something they do well either personally or professional.
**(2) Engaging in transparent and equitable resource sharing, cost charging, compensation, and incentives** Dr A discusses with Dr R what funds are available for the community members and for potential participant payments to collect additional data from Black and Hispanic patients to learn the reasons why this potential disparity may be occurring. Dr R informs Dr A what funds are available.

- Provide compensation for research team meetings attended and research activities performed that are aligned with the amount of time spent on the project

Dr A informs the community members how much they can be compensated monetarily for their efforts, including time spent in meetings and activities. Community members agree to the compensation. Dr A provides community members guidance on how to track time, who to submit time sheets to, and when to expect compensation. Dr A provides guidance on who to contact if compensation is not received in the expected timeframe.
**(3) Co-designing/co-conceptualizing the project** Prior to selecting policy implementation strategies, the project team needs to understand *why* this disparity may be occurring. The project team plans a formative evaluation specific to barriers and facilitators to policy implementation of measurement-based care for Black and Hispanic patients in the healthcare system.

- Assess the needs of the community and which impacts are most important to them

During the planning process, the community members suggest the project team also plan to visit the different community organizations and ask them why they think Black and Hispanic participants may be declining or not completing the measurement-based care measures. Dr A and Dr R attend and provide information about mental health, measurement-based care, and ask the organizations what they may want to know about measurement-based care. Different members of the organizations respond with the following concerns, “Why do these forms matter?” “Why is this important” “How do I know this is not going to be used against me?” “One of these questions is about suicide—how do I know I am not going to be locked up for responding to it?” The organization in general states they would want to know that these measures are safe for their members to complete and are useful for their members’ mental health care. Dr R stated he is confused and uncertain about why the community members did not understand the importance of measurement-based care when the two academics had just presented the information to them. Dr A stated it was important for the two of them as academics to hear their concerns and find out ways to relay the information in a manner that is acceptable and accessible to the community members. She informs Dr R that including the community members on the implementation team could be a path forward. She also suggests accessing the healthcare system’s plain language writing team to help draft the information may be ideal.
**(4) Co-refining the project and co-selecting strategies for project completion** Dr A presents the barriers and facilitators to the implementation team. She also plans to ensure that the policy implementation strategies that are selected for the project are acceptable to all members of the project team which includes researchers, healthcare system administration, health care providers, staff, and community members.

- Provide community members with information about implementation strategies

In addition to giving an overview of the barriers and facilitators, Dr A gives an overview of different policy implementation strategies. She also provides an overview of the ERIC taxonomy. She engages the implementation team in a Nominal Group Technique process [28] to vote for policy implementation strategies to ensure that everyone gets an equal vote. The Nominal Group Technique process is a consensus voting process in which everyone has an equal number of votes. It allows for equal distribution of power across all members of the group.
**(5) Bi-directional learning and open channels of communication during the project period** After the implementation team has conducted their formative evaluation and selected policy implementation strategies, they wish to inform community organizations about what they have learned and their next steps and to gather any additional input or concerns from community members.

- Provide presentations or infographic research updates

The implementation team works on a presentation of the barriers and facilitators to implementing the measurement-based care policy for Black and Hispanic patients in the healthcare system. They highlight the barriers as concerns and provide a response to each barrier. They then discuss the selected policy implementation strategies. This presentation is reviewed by the healthcare system’s plain language writing expert to ensure that it is accessible. It is also reviewed by several community members. Drs. A and R give the presentation at several of the community organizations that they visited. They ask if the community members have any additional input, concerns, or questions. They ask if there is anything the community members particularly want to know about the project. The community members in general respond that they believe they understand measurement-based care better now and would like to learn more about it in future presentations.
**(6) Taking action on harmful or inequitable policy implementation research and results** Based on an initial analysis of data, the implementation team learned the policy implementation effort was not being implemented equitably.

- Consider ways to change policy implementation research to alleviate harm or increase equitable implementation of policy

Dr R acted and reached out to an expert in implementation research and health equity who was able to help and now they will continue to measure throughout the process to assess if the health disparity has decreased. It is important to assess for potential differences by groups and conditions to examine if potential disparities are occurring in a policy implementation effort.

Case Vignette 3. CBPR in conducting policy impact evaluations
**Dr L and Ms H evaluate state-wide policy to distribute menstrual cups to unhoused women** Dr H is a researcher at State University Medical School who has partnerships with several non-profit organizations within the state who support unhoused women. A recent state-wide policy went into effect that these non-profit organizations will receive menstrual cups and funding to support implementing the policy. Dr H partners with six of the non-profit organizations to conduct a policy impact evaluation of the state-wide policy at their organizations.
**(1) Building or maintaining meaningful partnerships** Dr H has worked with these organizations for several years so the women who are regularly involved with the organizations are familiar with him. He also attends events for the organizations, he and his research group volunteer for the organizations, and some people involved with the organizations have gone on to become his graduate students.

- Include community members in the evaluation in ways that are acceptable and appropriate to them

One woman (Ms L) has worked with Dr H on several projects is interested in participating as part of the project team. She requests to co-lead focus groups with Dr H, which she has done before. This is also helpful for Dr H as she can relate to the unhoused women in a way that he cannot given her shared experiences with them.
**(2) Engaging in transparent and equitable resource sharing, cost charging, compensation, and incentives** Ms L is also currently unhoused and uses supports from the non-profit organizations so she asks Dr H about payment for working on the project.

- Provide compensation for research team meetings attended and research activities performed that is aligned with the amount of time spent on the project

Dr H states he can provide compensation for meetings, qualitative interviews, and informs her how to track her time for other activities. He can provide compensation through project funds and will need her to sign up for the medical university’s direct deposit service. Ms L reminds Dr H that she does not have a bank account and will need physical checks. Dr H contacts his department’s fiscal department who state they can create checks, but Ms L will need a permanent mailing address to mail them, which she does not have. Dr H speaks to the non-profit organization that Mr. L utilizes and they agree to serve as an intermediary to have checks sent there for her.
**(3) Co-designing/co-conceptualizing the project** Dr H is designing the policy implementation evaluation and is interested in several constructs to identify the impact of the policy on the state. Dr H uses the RE-AIM framework to design his policy impact evaluation. He is interested in measuring how many unhoused women receive menstrual cups (Reach), if the non-profits are successful in passing them out and educating the unhoused women who access their services (Adoption), acceptability and feasibility of using the menstrual cups (Implementation), and if the non-profits continue to purchase them and pass them out after one year (Maintenance).

- Assess the needs of the community and which impacts are most important to them

Dr H meets with several women who access services through the non-profits and asks them what they are interested in related to the menstrual cups. Their responses are their own finances. One woman replies, “I do not want to have to choose between food and period products. I hope this helps with that.” Dr H include the potential of reduced overall menstrual product cost to the women as a measure in the evaluation (Effectiveness).
**(4) Co-refining the project and co-selecting strategies for project completion** To guide selection of potential implementation strategies, Dr H conducted formative work to understand barriers and facilitators to policy implementation. Dr H presents the formative work to the non-profits, his implementation team, and community members for member checking. Community members respond that their biggest concern is that they will not know how to use the menstrual cup.

- Include community members in testing of implementation strategies

After reviewing the formative evaluation, the implementation team decides that they will use champions within the non-profit organizations as the implementation strategy. The strategy will be delivered with two options: (i) staff members of the non-profit organization as identified champions, (ii) community members as identified champions.
**(5) Bi-directional learning and open channels of communication during the project period** Throughout the process of the project, Dr H has been gathering feedback from the non-profit organizations, community organizations, and community members. He now has results from the formative evaluation and has planned the implementation impact evaluation. He wishes to present the results and next steps to community members.

- Provide research project results to community organizations in a way that is accessible

Dr H receives feedback from community members that several different methods of having access to the information is helpful for unhoused women because they may only be in a certain location temporarily or they may lose access to their phone or to the internet. The implementation team creates an infographic that is brief describing the barriers, facilitators, and next steps for the menstrual cup policy. Dr H makes the infographic available online through a QR code and has physical copies placed in the non-profit organization waiting rooms. Dr H and Ms L also give brief presentations at the community gatherings held by the non-profit organizations about the project so far.
**(6) Taking action on harmful or inequitable policy implementation research and results** After the policy had been implemented for one year, Dr H and Ms L began conducting focus groups for the policy impact evaluation. During the first focus group, they learn that several of the women became ill because they did not have access to a private washroom; a mild, unscented oil-free soap; or boiling water, all of which are necessary to clean and sanitize the menstrual cup. They also found that if they lost the bag it arrived in, they did not have a clean place to store it. These hygienic issues resulted in several vaginal infections and yeast infections. Dr H and Ms L were very upset by finding out this information and debriefed after the focus group.

- Inform the team of the harm caused and discuss with community members different implementation strategies that are focused on equity

Dr H brought this information to the non-profit organizations that he was partnered with and his implementation team. The implementation team and non-profit organizations collaboratively changed the implementation plan to include hygiene care and changed the funding in the implementation plan to include funds for soap, additional bags, and included handouts on nearby single-stall restrooms for the unhoused women to use to clean their menstrual cups. Dr H and Ms L also provided this information and resources to any additional participants in the evaluation.
